# Histopathological and Ultrastructural Changes after Electroporation in Pig Liver Using Parallel-Plate Electrodes and High-Performance Generator

**DOI:** 10.1038/s41598-019-39433-6

**Published:** 2019-02-25

**Authors:** B. López-Alonso, A. Hernáez, H. Sarnago, A. Naval, A. Güemes, C. Junquera, J. M. Burdío, T. Castiella, E. Monleón, J. Gracia-Llanes, F. Burdio, E. Mejía, O. Lucía

**Affiliations:** 1 Department of Electronic Engineering and Communications, University of Zaragoza, 50018 Zaragoza, Spain; 2 Hospital Clínico Universitario, 50009 Zaragoza, Spain; 3 Hospital del Mar, 08018 Barcelona, Spain; 4 Faculty of Medicine, Institute for Health Research Aragón, Zaragoza, Spain

## Abstract

Irreversible electroporation (IRE) has gained attention as a new non-thermal therapy for ablation with important benefits in terms of homogeneous treatment and fast recovery. In this study, a new concept of high voltage generator is used, enabling irreversible electroporation treatment in large tissue volume using parallel plates. Unlike currently available generators, the proposed versatile structure enables delivering high-voltage high-current pulses. To obtain homogeneous results, 3-cm parallel-plates electrodes have also been designed and implemented. IRE ablation was performed on six female pigs at 2000 V/cm electric field, and the results were analysed after sacrifice three hours, three days and seven days after ablation. Histopathological and ultrastructural studies, including transmission and scanning electron microscopy, were carried out. The developed high-voltage generator has proved to be effective for homogeneous IRE treatment using parallel plates. The destruction of the membrane of the hepatocytes and the alterations of the membranes of the cellular organelles seem incompatible with cell death by apoptosis. Although endothelial cells also die with electroporation, the maintenance of vascular scaffold allows repairing processes to begin from the third day after IRE as long as the blood flow has not been interrupted. This study has opened new direction for IRE using high performance generators and highlighted the importance of taking into account ultrastructural changes after IRE by using electron microscopy analysis.

## Introduction

**Figure 1:**
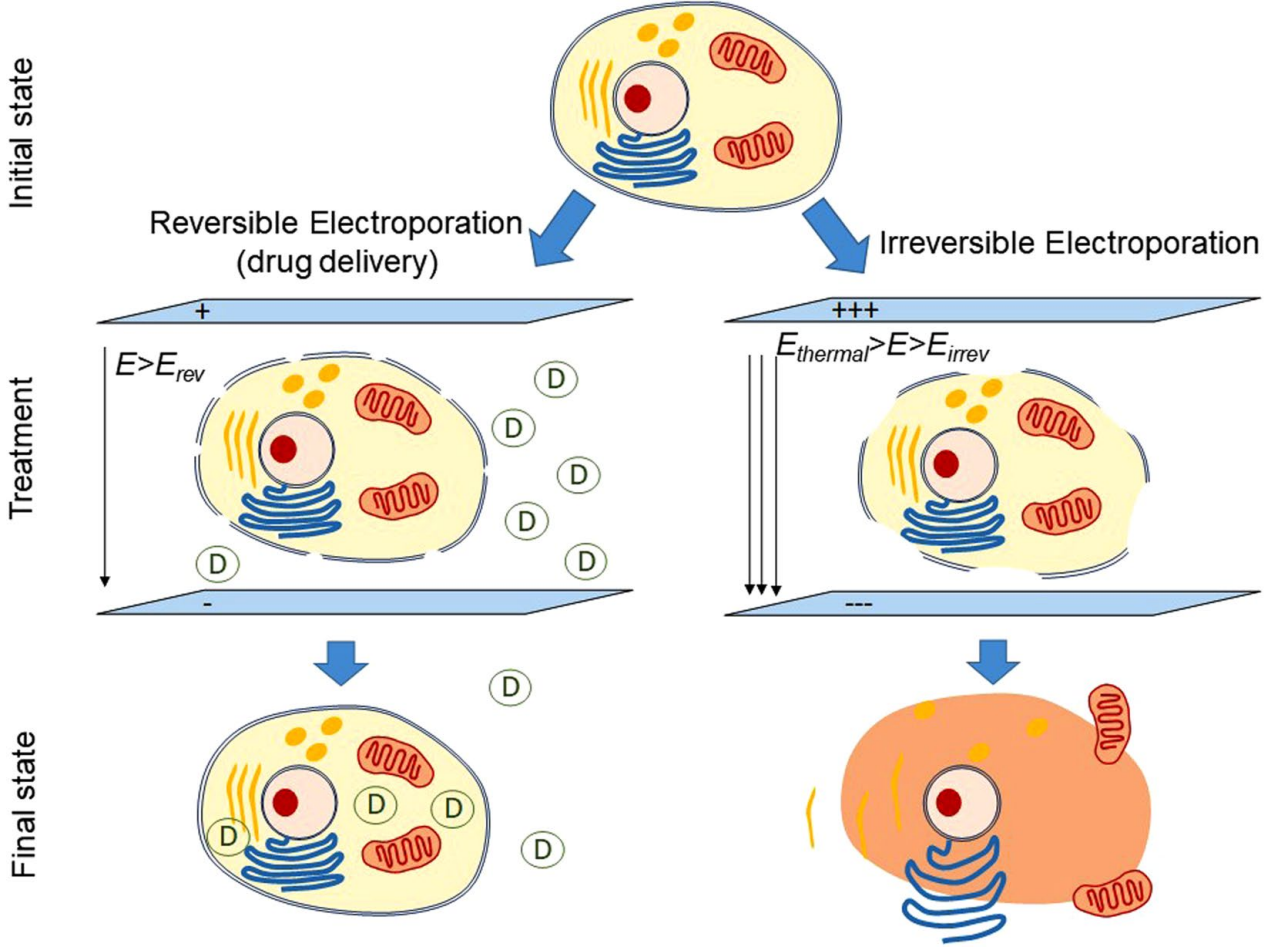
Electroporation. Reversible (RE, left) and irreversible (IRE, right) processes.

Electroporation (EP) is a technique based on applying high electric fields to increase cell membrane permeability^1^. Depending on the intensity of the field and the nature of the tissue, these effects can be temporal, i.e. reversible electroporation, RE, or permanent, i.e. irreversible electroporation, IRE (Fig. 1). In recent years, IRE has arisen as a promising ablation technique with potential to be an alternative cancer treatment for a variety of tumors such as hepatic, pancreatic, renal, prostatic or pulmonary tumors^2–4^. Its main benefits rely on the use of a non-thermal ablation source, which reduces thermal injuries, provides short-time treatments and enables the treatment of highly irrigated areas. This has led to a wide variety of clinical studies analyzing its effects to optimize the treatment^5^.

Tissue damage after IRE is a dynamic process with remarkable differences depending on the experimental conditions applied (characteristics of the electrodes used, voltage, pulses…), animal model, time between IRE and histopathogical examination, and applied study techniques. Typically, the applied electric field is in the range of 500–2000 V/cm, and the pulse length is in the 100 µs range to achieve the desired electroporation phenomena^5^. The pulse repetition rate is commonly below 1 Hz to avoid any thermal damage. Although many investigations have been published during the last years, there are very few that show the evolution of the lesions on liver and during a long enough period that permit regeneration of structures^6,7^. Most of them use pathological results based on histologic and immunohistochemical studies^8^, and not on ultrastructural studies. Transmission electron microscopy (TEM) studies of electroporated tissue are very scarce, and virtually non-existent scanning electron microscope (SEM) studies^9^. To our knowledge, there are two studies that describe TEM findings in uterine cervix and breast in rabbits^10,11^ and a study in pig liver which uses TEM methodology after IRE using needle but not parallel-plate electrodes^12^.

It is thought that the primary mechanism of cell death from IRE is apoptosis (programmed cell death), in contrast to coagulative necrosis (death by ischemic infarction), and both necrosis and apoptosis are likely to occur. However, the exact molecular mechanism of cell death after IRE is unknown^13^. So far it has not been considered that IRE may be involved by new form of cell death through a regulated necrosis known as necroptosis. Necroptosis is associated with rapid plasma membrane permeabilization, process that takes place in the IRE^14^.

Currently, many experimental IRE ablation techniques use pig models to assess the effect in the liver^15^. However, those studies are commonly limited by microscopy techniques applied^16^ and, specially, the available voltage generators^17,18^. In this paper, a detailed analysis of the temporal evolution of IRE effects using optical, TEM, and SEM microscopy is performed. The study will analyze the evolution after 3 hours, 3 days, and 7 days focusing on ultrastructural changes. Moreover, a newly developed high-performance high-voltage generator will be applied using 3-cm parallel plates to ensure homogeneous electric field, enabling higher intensity electric fields and treated areas.

## Materials and Methods

### Electrodes

**Figure 2:**
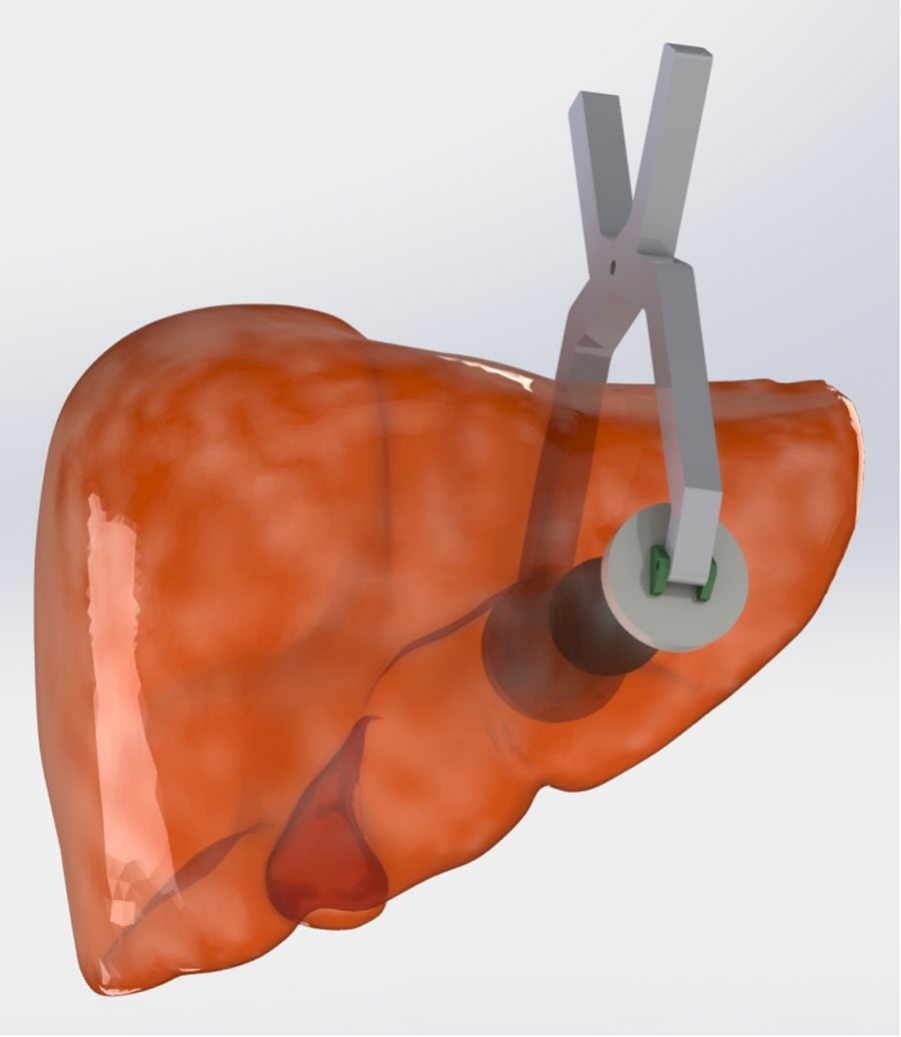
Example of application of IRE electrodes on human liver tissue using parallel-plate electrodes. “BodyParts3D liver” by Lambchops is licensed under CC-BY SAExample of application of IRE electrodes on human liver tissue using parallel-plate electrodes 2.1JP (This image has been modified).

**Figure 3:**
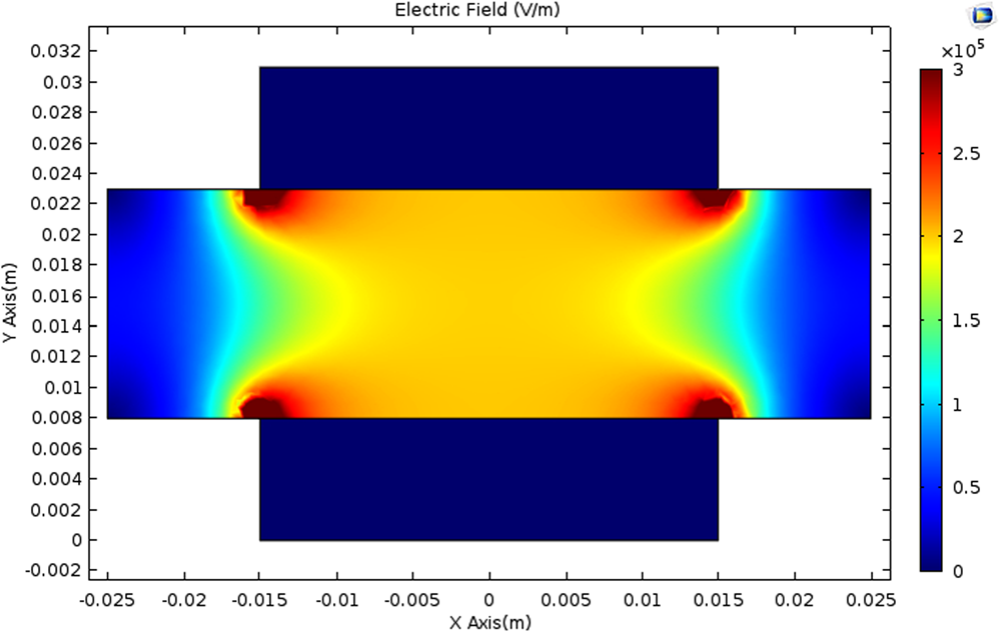
Electric field generated using 3-cm parallel plates. Electrodes are situated in the upper and lower part, and liver tissue is in the middle. Homogeneous field distribution is found in the central part, providing known and repeatable results for this study. Simulated with COMSOL Multiphysics v. 5.2, Stockholm, Sweden.

Electrodes are responsible for creating the required electric field in the region to be electroporated (Fig. 2). In order to obtain accurate results under homogeneous electric field, this study is carried out using circular 3-cm stainless-steel parallel-plate electrodes (Fig. 3). The electrodes were manufactured by the Group of Power Electronics and Microelectronics (GEPM) of the University of Zaragoza (Zaragoza, Spain). The electrodes were encased in a clamp, electrically isolated and connected to the high voltage generator.

### High-voltage generator

**Figure 4:**
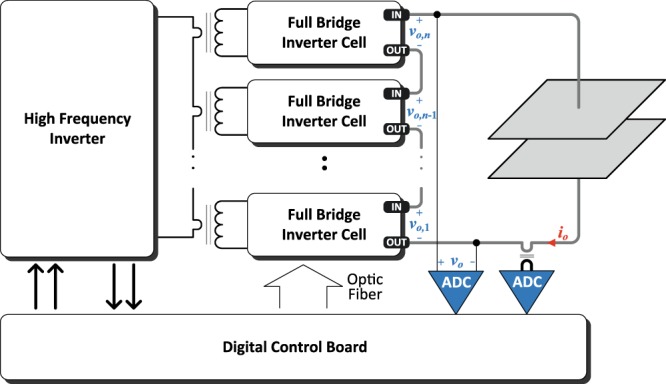
Versatile high-voltage pulse generator. The combination of isolated output full-bridge inverter cells provides high-voltage capabilities.

The high-voltage generator provides the required voltage and current to deliver the IRE treatment. The former ensures the required electric field, whereas the latter is proportional to the area to be treated. Currently, most commercially available devices are limited in voltage and current ratings, compromising research in this area. This research uses a versatile high voltage generator^19^ based on an isolated modular approach (Fig. 4) that enables achieving higher voltage and current. The power converter is controlled using a versatile field-programmable gate array (FPGA) control architecture to provide fully customizable IRE treatments, and it is designed to deliver up to 20 kV and 400 A, enabling research in new areas of IRE applications. Besides, each output full-bridge inverter cell is implemented by using a full-bridge inverter topology. This enables to generate arbitrary waveforms, including bipolar pulses which are essential to minimize electrolysis during electroporation. In this design, 1.7-kV silicon carbide power devices from Cree (Durham, NC) are used to provide high voltage and fast-switching capabilities.

**Figure 5:**
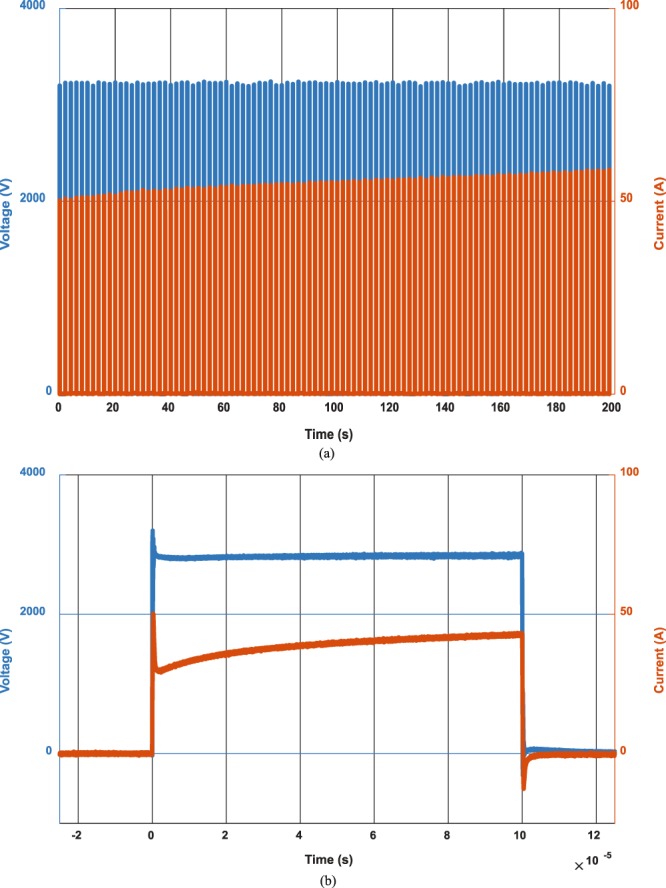
Main current and voltage waveforms during IRE treatment. Current and conductivity increases due to cell permeabilization. Full treatment train of pulses comprising 100 pulses of 100 µs (**a**) and detail of one pulse (**b**). Voltage polarity is changed between pulses and absolute values are plotted for effective representation. Besides, gap between pulses has been omitted for illustrative purposes.

Figure 5 shows an example of the main current and voltage waveforms during the experiments using 3-cm parallel plates and 3000 V in order to achieve 2000 V/cm electric field. In this figure, it can be seen the dynamic current increase due to cell permeabilization during the process^20^. To ensure the proper electric field application, the voltage applied in the electrodes has been directly measured using a high-voltage differential probe HVD3605 and MDA800 12-bit oscilloscope from Teledyne LeCroy (Chestnut Ridge, New York, USA).

### Animal model

The study was conducted in the facilities of the Institute of Health Sciences (IIS Aragón), Spain. A total of 6 healthy female pigs (Large white × landrace) were included in the experiment. We conducted the experiments in female pigs due to anatomical considerations, i.e. location of the penis in the middle line in male pigs which difficult the laparotomy. They were obtained from a usual supplier designed facility (Inga Food, Nutreco. Zaragoza, Spain), with an average weight of 45 kg. All the procedures were approved by the Animal Experimentation Ethical Commission, University of Zaragoza (permit number: PII19/16). All experiments were performed in accordance with relevant guidelines and regulations.

### Anesthesia

The surgical procedure and IRE delivery were performed under general anesthesia with oral intubation, mechanical ventilation and neuromuscular blockade to ensure complete muscular relaxation. The animals received premedication with tiletamine + zolazepam (Zoletil, Virbac, 0.05 mg/kg) and intramuscular Dexmedetomidine (Dexmopet, Fatro Iberica SL, 0.08 ml/kg). For anesthesic induction disopropilfenol (Propofol 1% MCT, Fresenius Kabi Laboratories Spain, 6 mg/kg maximun) is used, and for the maintenance we use sevofluorane 1.9% (Baxter SL). Muscular blockade was achieved with pancuronium bromide (Pavulon, Organon Española SA, 4 mg/2 ml). For the intraoperative analgesia we use iv fentanyl perfusion (Fentanest, Aurovitas Spain, 10 μg/Kg/h) and ringer-lactato (8 ml/kg/h) was the solution used as fluid therapy. Conventional ECG monitoring without connection between the monitor and the IRE source was used.

### Surgical procedure

A middle line laparotomy was performed to expose the entire abdomen. The liver was detached from adhesions to the diaphragm and the four hepatic lobes were mobilized for a proper exposition, every single lobe was isolated using cotton gauzes. The area of IRE treatment was selected in the distal part of every lobe, medial and lateral left and lateral right lobes were selected for the experiments, avoiding the medial right lobe.

After surgery animals are stabled in special conditions in heating cages. We use buprenorphine (Buprenodale, Dechra, 0.05–0.1 mg/kg/day) during the first 72 hours after surgery, and antibiotic prophylaxis with enrrofloxacine (Enroflox, Agrovet Market SA, 2.5 mg/kg) until the sacrifice. We daily observe the animals in order to detect problems with movement, behavior, appearance, intake and faeces, according to the established procedures. We also evaluate the surgical site.

### IRE settings

**Figure 6:**
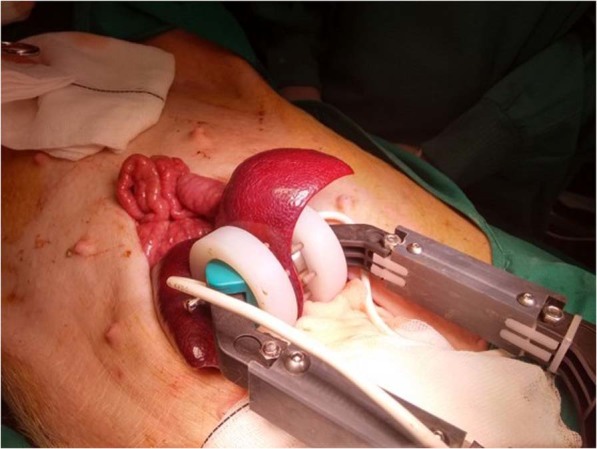
Experimental test. 3-cm stainless-steel parallel-plate electrodes placed embracing a lobe of the pig liver. Histological samples obtention. Three samples are obtained from the electroporated area (1), interphase with normal not treated tissue (2) and healthy tissue (3).

Two identical stainless-steel 3-cm-diameter circular plate electrodes (GEPM, Zaragoza, Spain) were placed parallel opposite embracing the selected hepatic lobe, target area (Fig. 6) was between the two plate electrodes.

The electrodes were located in the same position of every lobe of the liver in each animal to avoid variability. According to previous studies, electric field of 2000 V/cm was applied to ensure IRE using 100 pulses of 100-µs width with a separation to avoid thermal effects of 2 s. The distance between electrodes is measured at each location and the output voltage of the versatile high-voltage generator is adjusted consequently. Polarity is changed between pulses to avoid electrolysis.

Voltage and current data were recorded and stored in an oscilloscope. A probe located in the surface of the liver, close to the target area was used for temperature monitoring during the experiment to ensure that thermal coagulation do not occur. ECG and relaxation monitoring were achieved by an on-site anesthesiologist.

Three set of experiments were used: IRE application with sacrifice and sample collection at 3 hours, 3 days, and 7 days after the experiment, respectively.

### Sampling

The study has considered two animals per time of taking samples (3 hours, 3 days, 7 days), with 6 pigs in total. Open biopsies were obtained at the end of the procedure. The electroporated lobe was removed within the surgery and 2 parallel 0.5 cm thick slices were immediately collected from the center of the electroporated area. One slice was 10% formalin-fixed for histopathological analysis and the other slice was immersed into 2.5% glutaraldehyde (EMS, Hatfield, PA, USA) in 0.1 M phosphate buffer (PB) pH 7.4 for 4–6 hours for ultrastructural studies. Several glutaraldehyde-fixed pieces (1.5 mm3 for TEM and 5 mm2 for SEM) were trimmed and post-fixed overnight with 2.5% glutaraldehyde in PB at 4 °C.

In addition, control samples from normal not ablated tissue (from the same lobe) were collected and fixed for histopathological and ultrastructural analysis described previously.

### Sacrifice

At the end of the experiment or when issued (3 hours, 3 days, 7 days), after taking the samples for histological analysis, the animals were sacrificed using a potassium chloride injection (1 mEq/kg).

### Histopathological analysis

Formalin-fixed tissues were trimmed and processed according to standard histopathological procedures. A 5 µm tissue section from each sample was stained with haematoxylin and eosin (H&E) for light microscopic examination (Olympus BX51 microscope, Olympus Imaging Corporation, Tokyo, Japan). Electroporated area was evaluated by two skilled pathologists who assessed lesions and regeneration signs and subjectively scored based on their extent and frequency (Table 1).

**Table 1:** Qualitative result summary of the optic and electronic microscopy analysis.

	3 hours	3 days	7 days
Lobular architecture preservation	+++	++	+
Hepatic plates organization	+++	++	+/−
**Lesions**			
Hemorrhage	+++	+	−
Inflammation	−	+	++
Sinusoidal endothelium	−	−	−/+
**Hepatocytes**			
Plasma membrane disruption (EM)	+++	+++	+++
Plasma membrane disruption (OM)	+	++	+++
Cellular discohesion (OM)	+	++	+++
Nuclear changes (OM)	+	++	+++
Nuclear envelope preservation (EM)	+++	+	−
Vessel endothelium	−	+/−	++
Bile duct epithelium	−	++	+++
Necrosis signs	+	++	+++
**Regeneration Signs**			
Spindle cells	−	++	+++
Mitotic stromal cells	−	+	+++
Multinucleated hepatocytes	−	++	+++
Fibrosis	−	+	+++
Angiogenesis	−	+	+++
Regenerative nodules	−	+	+++
Hepatic acinar organization	−	+	++
Neoformation of portal bile ducts	−	++	+++
Mitotic bile duct epithelium	−	+	+++

(−): Not observed; (+): Focal or scarce; (++): Frequent; (+++): Very frequent.

EM: electron microscopy; OM: optic microscopy.

**Figure 7:**
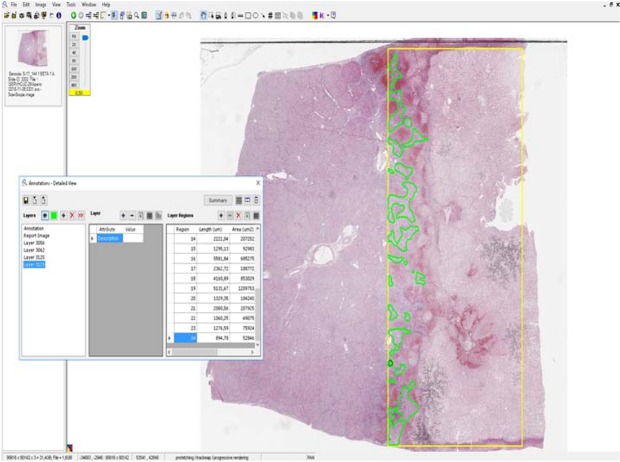
Digital image analysis of the electroporated area. The measured area is in the yellow box (165 mm2) and encompasses the necrotic area. The green zone corresponds to the regenerative zones.

Images of each whole tissue section were acquired by a slide scanner (Aperio AT2, Leica). By digital image analysis (Aperio ImageScope- Pathology Slide Viewing Software, Leica), a 165 square millimeter rectangle from the middle of each electroporated zone (from the dorsal to the ventral fibrous capsule) was selected (Fig. 7). Within this rectangle, necrosis/regeneration areas were measured, and subsequently results were presented as a percentage.

### Transmission electron microscopy (TEM)

Tissue samples were washed with PB, post-fixed with 2% osmium tetraoxide (EMS) in PB for 2 hours at room temperature, dehydrated through graded ethanol series (30%, 50%, 70% with 2% uranyl-acetate for 2 h, 90%, 100%) cleared in propylene oxide (Fluka AG, Switzerland) and then, embedded in araldite (Durcupan, Fluka AG). Finally, araldite was polymerized overnight at 60 °C. The resulting blocks were cut on serial ultrathin sections (80 nm) using a Leica (Nussloch, Germany) EM UC6 ultramicrotome, collected on Formvar-coated single-slot grids and counterstained with 1% uranyl acetate and Reynold’s lead citrate for 10 min. They were examined by a JEOL JEM-1010 transmission electron microscope (Tokyo, Japan).

### Scanning electron microscopy (SEM)

After fixation, the samples were then washed thoroughly with PB, post-fixed with 1% osmium in PB for 2 hours and dehydrated through a graded ethanol series. For drying, the samples were immersed for 30 min. in a mix of 50% hexamethyldisilazane (HMDS, EMS) and 50% ethanol, and finally 100% HMDS for 30 min. After HMDS treatment, the samples were removed from the wells and excess HMDS was blotted away by filter paper. The samples were then transferred to a desiccator for 20 min. to avoid water contamination. After drying, the samples were mounted onto an aluminum metal plate and coated with a thin layer of gold. Samples were examined with a JEOL JSM 6360-LV (Tokyo, Japan) microscope.

## Results

### Optical microscopy (H&E)

**Figure 8:**
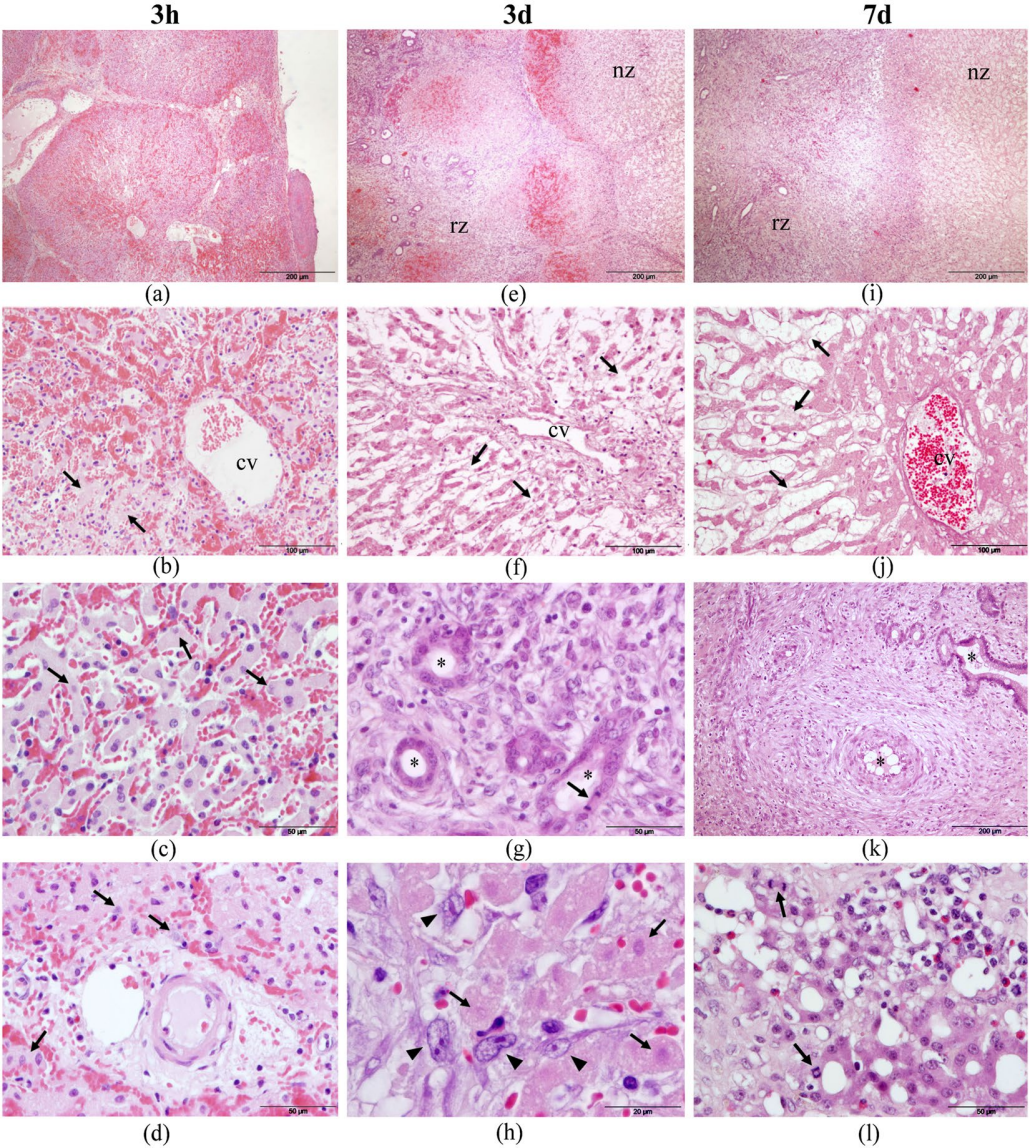
Histopathology of the liver after IRE ablation with H&E stain. Three hours (3 h) after IRE the lobular architecture is preserved although with visible hemorrhagic, congestion, and edema(**a**–**d**). After three (3d) and seven days (7d), two zones can be distinguished: one necrotic (nz) and the other with signs of cellular regeneration (rz) (**e**,**i**). The hepatocytes of the electroporated area appear irregular and with disfigurement of the membranes, apparently intact nuclei although some of them with karyolysis (arrows, (**c**,**d** and **h**)). The centrilobular vein (cv) and the hepatocyte trabeculae are distorted with loss of hepatocytes (arrows) and endothelial cells (**b**,**f** and **j**). The reparative area show: new bile ducts and vessels (asterisks, (**g**,**k**)); mesenchymal spindle cells infiltrating the necrotic area (arrowheads, (**h**)) and regenerative nodules of hepatocytes with acinar arrangement and mitotic figures (arrows, (**l**)).

Three hours after IRE, the lobular architecture appears conserved although with marked disruption of the hepatic parenchyma produced by an extensive hemorrhage of variable intensity according to the areas (Fig. 8(a)). The trabeculae of hepatocytes are distorted by the hemorrhage that occupies and distends sinusoidal and perisinusoidal spaces. Disappearance of the endothelial lining of the sinusoids and shedding of pericentral hepatocytes are also evidenced (Fig. 8(b)). The hepatocytes appear discohesive with irregular cell borders and membrane defacement/damage as incipient signs of cell dead. Most nuclei apparently remain intact, although some of them present pyknosis and karyolysis (Fig. 8(c)). At septal and portal space levels, there is hemorrhage and edema with dilated vessels presenting not conserved endothelium (Fig. 8(d)). Bile ducts show altered epithelia.

Analyzing the liver tissue of those animals euthanized after three days, lesions observed can be differentiated into two well recognized zones (Fig. 8(e)):

1. The electroporated area that presents evident cellular necrosis although the lobular architecture is conserved. The centrilobular vein appears altered with loss of endothelial cells. In the septal and portal space, congestion and thrombosis in some blood vessels were observed. The hepatocytes already show clear signs of alteration as seen at three hours (Fig. 8(f)).
2. Areas of hepatocyte necrosis and hemorrhage coexist with those presenting reparative signs. The septa appear enlarged showing proliferation of mesenchymal spindle cells that will give rise to bile ducts of active epithelium with mitosis figures (Fig. 8(g)). Vascular proliferation with prominent endothelia has also been observed. The regeneration of hepatocytes is initially manifested by the presence of spindle cells that penetrate from the periphery to the center of the necrotic lobule (Fig. 8(h)). Nodules of hepatocytes with newformed appearance and proliferative signs such as presence of mitosis and even sometimes multinucleation are recognized.

Seven days after IRE, the two zones still could be differentiated:

1. An electroporated area with easily detectable necrosis of the lobules and in some vessels.
2. An adjacent area in which marked reparative and inflammatory signs appear more evident than at three days after IRE (Fig. 8(i)). The proportion of eosinophils was remarkable (Fig. 8(j)). In this last area, a marked proliferation of mesenchymal spindle cells around foci of necrosis, giving rise to numerous bile ducts and newformed vessels are observed (Fig. 8(k)). These same cells result in regenerative nodules of hepatocytes, which frequently have acinar arrangement and mitotic figures (Fig. 8(l)). The regeneration of hepatocytes is more prominent than that observed in euthanized animals three days after IRE.

Using ImageScope software, a 100% area of necrosis was observed in the electroporated selected area by 3 hours. A 94,62% and 46,69% areas of necrosis (with 5,63% and 51,40% of regeneration changes respectively) were observed by 3 and 7 days.

### Electron microscopy (SEM and TEM)

**Figure 9:**
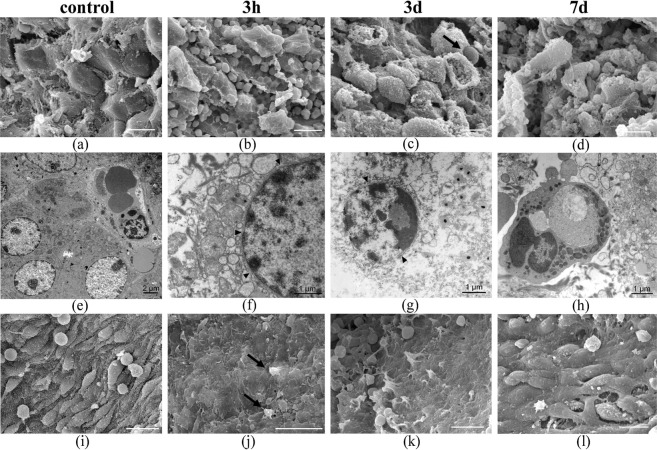
Electron micrograph of pig liver after IRE. Portion of a liver lobule showing plates of hepatocytes damaged but preserving its structure (**b**–**d**) 2,000X. Under TEM, the hepatocytes show lysis of the plasma membrane with the release of organelles, dilatation of the granular endoplasmic reticulum and mitochondrial alterations but the nuclear envelope is preserved (arrowhead, **f**,**g**). After 7 days (7d), phagocytic cells are frequent, phagocytizing cell debris (**h**). SEM of large blood vessel, after 3 hours (3 h) and 3 days (3d), illustrating the lack of endothelial cells and their substitution by platelets (arrows) (**j**,**k**) 2,000X. After 7 days, endothelial cells are distinguished in some vessels (**l**). Controls were taken in healthy tissue (**a**,**e** and **i**). Scale bar (**a**–**d**,**i**–**l**) 10 μm.

After three hours, the external shape of the hepatocytes dispersedly appears irregular without microvilli and a rough surface while non-electroporated hepatocytes show smooth surface with small microvilli (Fig. 9(a,b)). Sinusoid remnants are observed close to them. At these experimental conditions, TEM evidences variable degree lysis of the plasma membrane. In some hepatocytes, their plasma membranes disappear completely, while in others, small pores appear, coexisting with large areas of loss of membranes. Despite it, cellular shape remains. On the contrary, both membranes of the hepatocyte nuclear envelope remain conserved justifying the intact appearance of nuclei in many of them (Fig. 9(f)). Disruption of the plasma membrane also occurs in Kupffer and Ito cells. Cellular organelles also present different degrees of alteration that are manifested initially by very dilated saccule of endoplasmic reticulum rough and loss of mitochondrial crests (Fig. 9(f)).

Despite it, cellular shape remains, perhaps due to a coagulation process of the cellular material. On the contrary, both membranes of nuclear envelope of the hepatocytes remain conserved justifying the intact appearance of nuclei in many of them (Fig. 9(f)). Disruption of the plasma membrane also occurs in Kupffer and Ito cells.

After three days, the rough shape of the electroporated hepatocytes continues to be recognized beside organelles and isolated nuclei (Fig. 9(c) (arrow)).

**Figure 10:**
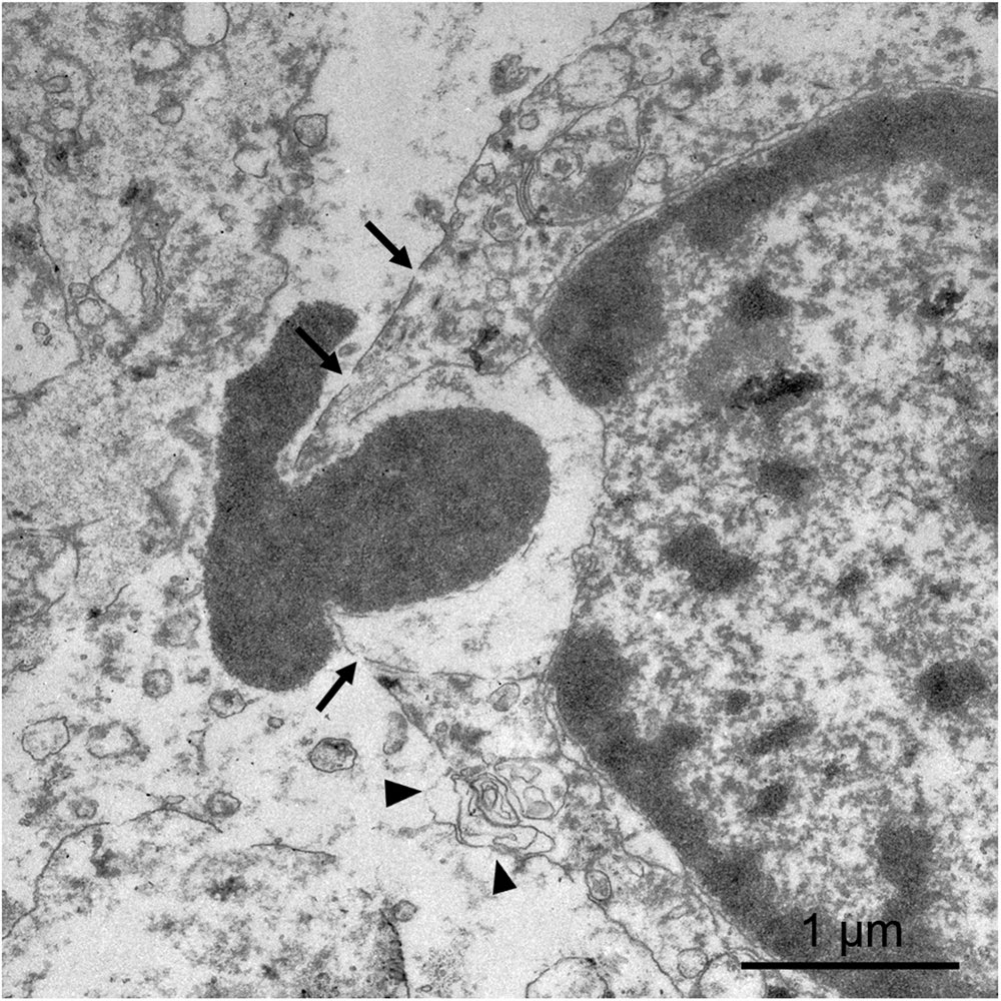
TEM 3 days after IRE. The plasma membrane of the hepatocytes shows small pores (arrows) together with large areas in which the membrane has disappeared (arrowheads). Amounts of chromatin are expelled from the nucleus to extracellular space.

In some hepatocytes, small pores (20–50 nm diameter) are observed coexisting with large areas in which the plasma membrane has disappeared, resulting in the release of cellular organelles and even amounts of chromatin (Fig. 10). These ultrastructural observations are compatible with cell death by a necroptosis process. Neither endothelial cells are recognized, although thrombi can be observed adhered to the inner surface of blood vessels in the electroporated area (Fig. 9(k)).

Seven days after electroporation, numerous degenerated pleomorphic hepatocytes presenting deep pores and irregularity in their surface (Fig. 9(d)). In the necrotic lobules of the electroporated area, abundant phagocytic cells are found, cleaning cellular debris by engulfing them inside their phagosomes (Fig. 9(h)). Three types of vessels could be distinguished in the necrotic area by SEM: some with thrombi in their lumen and signs of necrosis; others without thrombi and abundant leukocyte infiltration but without endothelial cells; and the others with groups of prominent endothelial cells recognized among cellular debris, platelets and fibrin (Fig. 9(l)). This last observation suggests endothelization process. These results are summarized in Table 1.

## Discussion

In this study, the effects of IRE using and advanced high voltage generator and parallel-plates to ensure uniform electric field exposure has been analyzed. Previous studies in pig liver cells^5^ describe the cell viability as a sigmoid function depending on the applied electric field. For pig liver cells, typically a minimum threshold for IRE is described at 600–800 V/cm, being the percentage for no cell viability established close to 2000 V/cm. However, these studies are frequently limited due to the used generators and they are performed either in cuvette or with lower voltage/current levels in *in-vivo* liver. In this study, however, by using the newly developed generator we have been able to apply 2000 V/cm in all the samples.

Under the experimental conditions used, histological analysis revealed acute tissue damage in the treated regions 3 hours after IRE. At that time, an extensive hemorrhage which causes a marked dilation of sinusoids, together with a wide cellular destruction including hepatocytes, Kupffer cells, Ito cells and biliary epithelium was observed. This is supported by previous publications^2,21,22^ that also described congestion in sinusoids and coagulative necrosis of hepatocytes three hours after IRE. It has been suggested that in conditions of high voltage, IRE can produce sufficient heating to induce “white zone” thermal coagulation^17^.

By electron microscopy, this tissue damaged can be explained by alterations of the plasma and organelles membranes except the nuclear envelope which initially remains intact. By optic microscopy, hepatocytes exhibit preservation of the cell outline with a homogeneous and eosinophilic cytoplasm that may be due to the sudden coagulation of cell proteins. Previously, it has been suggested that hepatocyte death occurs by a process of apoptosis^23–28^, a regulated cell death characterized by preservation of the plasma membrane, until formation of cell fragments into membrane-bound particles (apoptotic bodies)^29^. Despite our study has revealed ultrastructural changes in some hepatocytes, such as nuclear chromatin condensation into a half-moon shape attached to the nuclear envelope which is characteristic of apoptosis, the plasma membrane disruption observed is not compatible with a process of apoptosis. An explanation for this event may be that apoptosis occurs when the electric field intensity applied is lower than the used in this study (1000–1500 V/cm)^16^. Another possibility consist of other type of cellular death is occurring in those cells.

Among the cellular death processes, necrosis has long been described as an instantaneous and uncontrollable cell death consequence of extreme physical, chemical, or mechanical stress. This accidental cell death is characterized by breakdown of plasma membrane, though nuclei remain largely intact during this process. Loss of membrane integrity implies release of intracellular content that induces an inflammatory response^14,30–32^. Necroptosis is a modality of regulated cell death triggered by perturbations of extracellular or intracellular homeostasis that is dependent on activation of receptor –interacting kinase (RIPK3) and the mixed lineage kinase domain-like (MLKL)^33^. In the present study, we observed by TEM that nuclear envelope initially remains intact (at least for 3 days after IRE), but is progressively disrupting until day 7 when is completely disrupted. In accordance with our observations, a recent molecular study found that RIPK3 and MLKL were elevated on IRE treated rat liver, indicating that necroptosis pathway was induced^7^. This phenomenon of necroptosis does not exclude the appearance of wide areas of necrosis and inflammatory reaction when increasing the time post IRE.

The present study shows that the endothelial cells of the blood vessels are also affected 3 hours after IRE. We observed a loss of the endothelium of sinusoids, centrilobular veins and large blood vessels although the architecture of the largest vessels is conserved and sometimes remain permeable to blood flow. This is consistent with other authors where electroporation treatments of 2500–3000 V/cm in liver hilum vessels were relatively preserved^34^. However, the apparent maintenance of the extracellular matrix of blood vessels is not accompanied at the cellular level. By electron microscopy, we observed a clear loss of endothelial cells from 3 hours to 7 days. In other types of experiments, IRE with 3800 V/cm have allowed to observe a clear decrease in smooth muscle cells but the endothelial cells were preserved after 28 days in carotid artery^35^. However, after 3 hours pulses of less electric field (1000 V/cm) but longer pulses (20-ms versus 100-µs), rats livers exhibited microvascular occlusion, endothelial cell necrosis and diapedeses^2^. Apart from the difference in the vessels size, surrounding tissues and electroporation feature, this could indicate that at the beginning, there is a rapid necrosis of endothelial cells that have regenerated after a few days. The interruption of the endothelial monolayer is rapidly (within hours or days) recolonized by endothelial cells, originating from replicating neigh-boring cells and/or circulating endothelial progenitor cells^36^. Platelets play a critical role in regeneration and, if they are activated, they release upon activation growth factors that are involved in endothelial cells proliferation, migration and differentiation of mesenchymal stem cells^37^. In fact, the platelets activation due to endothelial damage has been previously described in case of reversibly electroporated skin^38^. Focusing our attention in IRE, we have observed in the 3 hours experiment, that there is an intense activity of platelets in the luminal surface of the blood vessels of the electroporated area that disappears after 3 days. It has been observed that blood flow can be preserved in the electroporated area, even in small vessels. Therefore, the non-interruption of the blood flow with electroporation would allow the arrival of platelets to the injured area early after IRE and let the recovery of the endothelium of large vessels as we observed at 7 days. However, we have found that preservation of the vessels lumen is not a homogeneous finding since the formation of thrombi has been found in some instances.

One of the most interesting findings in the present study is the fast process of healing observed in the electroporated surrounding area. Three hours after IRE, mesenchymal-like cells are activated showing an undifferentiated appearance and voluminous nucleus; these cells proliferate, colonize the necrotic area and seem to regenerate the biliary epithelium and immature hepatocytes forming acinar structures. This result is in accordance with previous studies which reported that the intrinsic hepatic progenitor cells (HPCs) are bipotential and can regenerate both biliary epithelia and hepatocytes^39–41^. The process of healing is more extensive at 7 days after IRE. Most of the time-dependent effects of IRE studies focusing on liver report a regeneration of liver, but there is not consensus on when regeneration begins. Our findings are in accordance with Golberg *et al*.^6^, showing regeneration events at 3 days after IRE in rat liver.

## Conclusions

This study has proved the feasibility of the developed high voltage generator for homogeneous electroporation of large volumes of liver tissue using parallel plates. It has been proved that the experimental conditions used are ideal for achieving total destruction of the cells in the electroporated area.

The destruction of the membrane of the hepatocytes and the alterations of the membranes of the cellular organelles seem incompatible with cell death by apoptosis. Although endothelial cells also die with electroporation, the maintenance of vascular scaffold allows repair processes to begin from the third day after IRE as long as the blood flow has not been interrupted.

The ultrastructural study carried out has been key to be able to clearly define some of the biological processes described in our results, so we believe that electron microscopy studies should be included in studies carried out in preclinical IRE applications.

Future research lines should include the analysis of the electric field influence, as well as the different IRE modulation parameters. Besides, safety evaluation regarding the maximum current/voltage applicable should be done, and clinical translation to human liver tissue with tumors must be analyzed.
